# A Case of Polyarteritis Nodosa Presenting as Rapidly Progressing Intermittent Claudication of Right Leg

**DOI:** 10.1155/2017/4219718

**Published:** 2017-10-04

**Authors:** Chathuranga Lakmal Fonseka, Sampath Rukshani Galappaththi, Dayakshi Abeyaratne, Nirmali Tissera, Lalith Wijayaratne

**Affiliations:** ^1^Department of Internal Medicine, Faculty of Medicine, University of Ruhuna, Galle, Sri Lanka; ^2^National Hospital, Colombo, Sri Lanka; ^3^Internal Medicine, National Hospital, Colombo, Sri Lanka

## Abstract

**Background:**

Polyarteritis nodosa (PAN) is a medium vessel vasculitis which causes significant morbidity and mortality. Usually, it presents with constitutional symptoms with angiographic evidence of aneurysms or segmental stenosis of arteries of mesenteric or renal vasculature. It is exceedingly uncommon for PAN to present with symptomatic progressive intermittent claudication.

**Case Presentation:**

We describe a 60-year-old male who presented with rapidly progressive intermittent claudication of his right leg. He did not have any significant atherosclerotic risk factors. He had recent onset hypertension and loss of weight. He also had mononeuropathy of right common peroneal nerve and livedo reticularis rash. With negative autoimmune markers and suggestive histology in deep punch skin biopsy and angiographic evidence of segmental stenosis of femoral and renal arteries, we diagnosed PAN. We treated him with aggressive immunosuppressants and vascular bypass surgery of right femoral vessels; he showed a good response.

**Conclusion:**

Rapidly progressive unilateral intermittent claudication could be a very rare, but noteworthy presentation of PAN. With suggestive histology and exclusion of other comorbidities aggressive immunosuppressants should be instituted. Vascular bypass surgery for critical ischaemia of the limbs is an option that could be considered for limb-threatening disease.

## 1. Background

Polyarteritis nodosa (PAN) is a rare form of vasculitis predominant in the adult population. It is a systemic disease that typically affects medium-sized muscular arteries. Unlike some other forms of vasculitides, PAN is not associated with antinuclear or antineutrophil cytoplasmic antibodies (ANCA) [1]. The organs most commonly affected by PAN include the intestinal tract, kidneys, nerves, and joints. Respiratory and cardiac involvements are seen infrequently. PAN usually presents with constitutional symptoms, new onset hypertension, and elevated erythrocyte sedimentation rate (ESR) with absent autoimmune antibodies such as antinuclear antibodies, ANCA, and rheumatoid factor [2, 3]. One of the most suggestive features of the diagnosis is its predilection to the arterial territories in various organs of the body, mainly kidney and mesenteric arteries.

As a result of inflammation of these arteries, the vessel wall gets weakened and stretches leading to aneurysm formation or becomes thinned leading to rupture resulting in bleeding into the tissues. Vascular inflammation can also cause blood vessel narrowing leading to organ ischaemia.

CT angiography or MR angiography is commonly performed to demonstrate aneurysmal and nonaneurysmal changes including larger aneurysms and stenosis of the medium-sized vessels. Aneurysms are most commonly found in the kidney, liver, and mesenteric arteries and they occur mostly at the branch points [4, 5]. Aneurysms are usually seen in about 48% of patients, and segmental occlusive lesions (luminal irregularities resulting in reduction of caliber, stenosis, or occlusion) were seen in 30% of patients, and 13% had both the above abnormalities [6].

Out of the sites of vascular involvement in PAN, peripheral arterial involvement is exceedingly uncommon [3]. We describe a patient with PAN who presented with predominant peripheral arterial involvement presenting with unilateral rapidly progressing leg claudication.

## 2. Case Presentation

60-year-old previously healthy male presented with a progressive intermittent claudication of his right leg of 2-month duration. He is a nonsmoker and occasionally consumes alcohol. He did not have a past history of diabetes mellitus, hypertension, or dyslipidaemia. His right lower limb claudication gradually became worse over one month and he experienced right leg pain even on minimal exertion in walking few meters. For the last four months, he had an evening low grade fever, profound myalgia with loss of appetite, and a gradual weight loss of 8 kg. Two weeks before the admission he noticed a weakness of his right ankle with a rash on his right leg. He had no history of abdominal or testicular pain.

**Figure 1:**
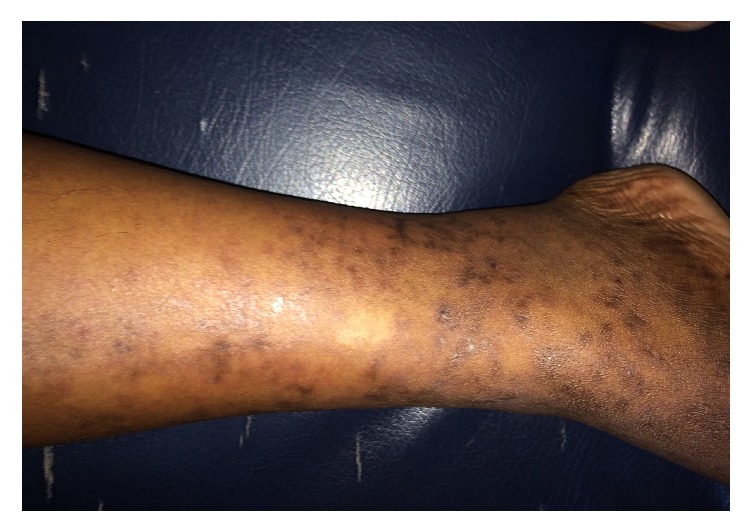
Livedo reticularis of right leg.

On examination he was afebrile, pale, and cachectic. There was no lymphadenopathy or organomegaly. He had a livedo reticularis rash over the right leg with polymorphic vasculitic changes (Figure 1). His blood pressure was 160/100 Hg mm. The examination of peripheral pulses revealed absent femoral, popliteal, and distal pulses of the right lower limb. On the left lower limb, he had preserved popliteal and femoral pulses with absent dorsalis pedis and posterior tibial pulses. His respiratory system and abdominal examination were normal. He had a right-side foot drop with sensory impairment over the right dorsum of foot with preserved deep tendon reflexes. Fundal examination was normal.

**Figure 2:**
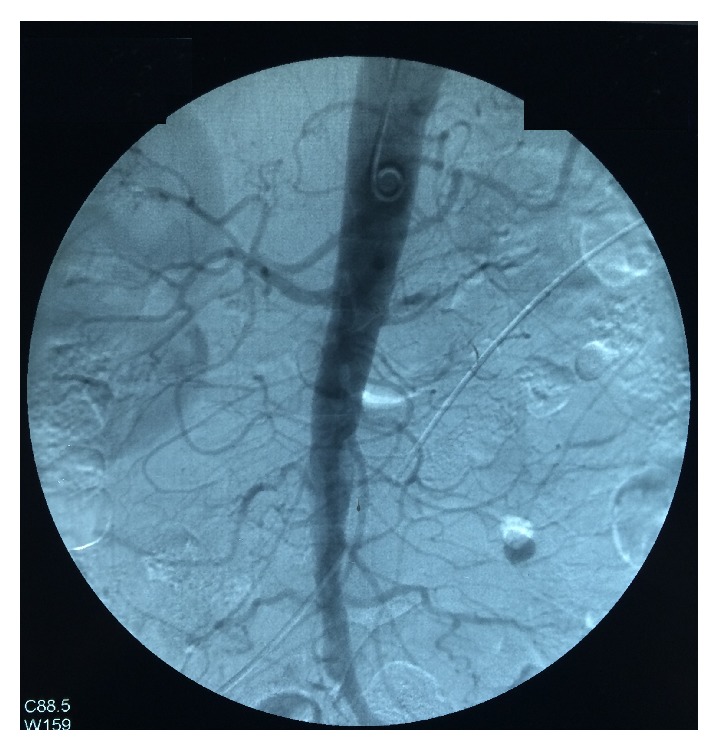
Digital subtraction angiography demonstrating left renal artery stenosis.

**Figure 3:**
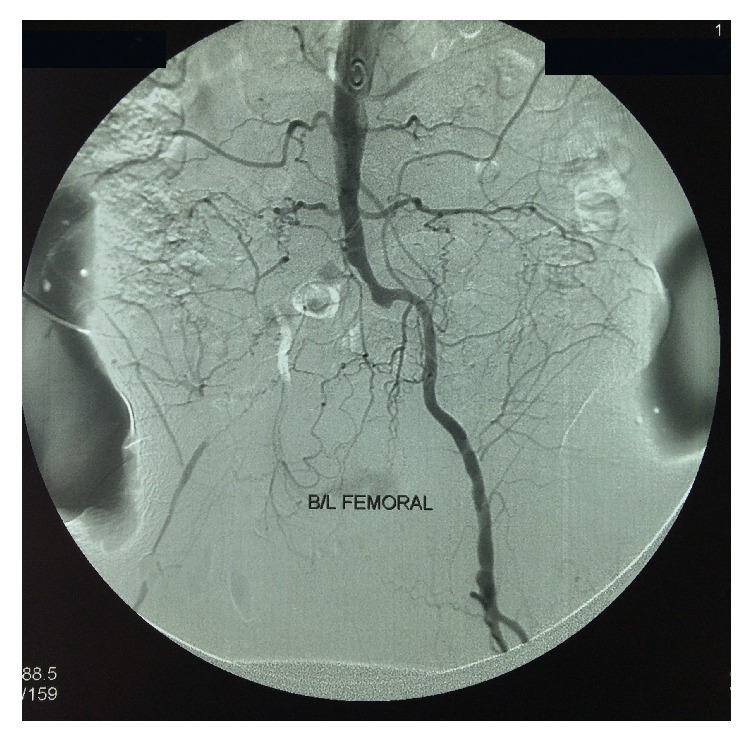
Digital subtraction angiography showing severe long segment stenosis of right-side iliofemoral arteries.

His investigations revealed normocytic normochromic anaemia (8.4 g/dl) with an ESR of 120 mm per 1st hour. His CRP was 92 u/l (<6). Transaminases were normal and his albumin to globulin ratio was reversed. Renal functions were normal (serum creatinine 90 mmol/l) and the urine tests revealed a significant microscopic haematuria without proteinuria or dysmorphic red cells. Chest radiograph was normal. His autoimmune antibody testing including ANA and ANCA (including MPO and PR3) and rheumatoid factor was negative. His 2D echocardiogram, homocysteine level, and HbA1c and lipid profile were within normal limits and the HIV status was negative. His hepatitis B and C status was also negative. Digital subtraction angiography of aorta and lower limb revealed left side renal artery stenosis (Figure 2) with segmental occlusion of left iliofemoral and right femoral arteries (Figure 3). There was no evidence of aneurysms in angiography. Nerve conduction study revealed right-side peroneal nerve palsy. Deep punch biopsy from vasculitic skin lesions showed segmental transmural inflammation of muscular arteries with fibrinoid necrosis with a polymorphonuclear infiltrate suggestive of a medium vessel vasculitis.

With the history of constitutional symptoms with severe myalgia, new onset hypertension, mononeuropathy together with angiographic changes, and medium vessel involvement in histology, we diagnosed PAN in our patient. After excluding viral causes such as hepatitis B, hepatitis C, and HIV, we had to start immediate immunosuppressive medication. Due to rapidly progressing limb-threatening disease, we started the patient on steroid pulses and intravenous cyclophosphamide. Subsequently, due to the disabling ischaemic rest pain we referred the patient to vascular surgical team and he underwent a successful right-side iliofemoral bypass.

After aggressive immunosuppression and bypass surgery our patient could mobilize with aid and his constitutional symptoms and the vasculitic rash improved with time. The rest of the pain of right lower limb disappeared and he could walk for 20–30 m without developing claudication. We continued giving the patient steroids tailing off gradually and planned on monthly intravenous cyclophosphamide therapy. With treatment, his constitutional symptoms and myalgia reduced and the ESR came down to 60 in 1st hour. His weight was stable. Within the next 6 months, while on immunosuppressants, he was admitted with recurrent infections to the hospital, so that we had to stop cyclophosphamide and switch to azathioprine.

## 3. Discussion

Peripheral vascular disease is not an uncommon finding in the general population. Usually it is slowly progressive associated with risk factors such as smoking, diabetes mellitus, and dyslipidaemia. They commonly present with symmetrical intermittent claudication of lower limbs. PAN is a necrotizing vasculitis involving medium to small vessels of most organs in the body, sparing the lungs, glomerulus capillaries, and the venous system [7]. It may rarely present as premature peripheral vascular disease usually in less-than-60 age group. As there are no specific diagnostic investigations of PAN, the diagnosis is clenched by considering the whole clinical picture with abnormal angiographic finding and biopsy findings of fibrinoid necrosis with predominant polymorphonuclear infiltrate suggestive of small to medium vessel vasculitis [8]. Exclusion of ANCA associated vasculitis such as microscopic polyangiitis (MPA) and atypical presentations of giant cell arteritis (GCA) which may give rise to similar presentations is important when arriving at the correct diagnosis. In our case, considering the age and biopsy characteristics, GCA becomes unlikely since it commonly gives rise to lymphocytic infiltrate with giant cells involving extracranial carotid arteries, usually in the elderly population. A negative ANCA, absence of evidence of glomerulonephritis, and lung involvement make MPA less likely.

There are several case reports of PAN among adults, who presented with rapidly progressive intermittent claudication [9–14]. All these patients had evidence of symmetrical progressive intermittent claudication of the lower limbs. Our patient presented with rapidly progressing predominantly unilateral intermittent claudication with asymmetrically absent pulses of lower limbs with high inflammatory markers and negative autoimmune markers such as ANA and ANCA. In one of the above cases, a patient has well responded to aggressive immunosupppression [13] and another patient has succumbed due to the side effects of the drug [12]. Our patient developed recurrent serious infections which made us discontinue his immunosuppression.

In the two cases reported by Ninomiya et al. [9] and Heron et al. [10], there were demonstrable characteristic microaneurysms. But, Shukla and Aggarwal [13] and De Golovine et al. [12] both described two cases with segmental stenosis of large vessels without aneurysmal dilatations, similar to our case. Our patient had unilateral predominant femoral involvement with unilateral renal artery stenosis. This may mean that segmental stenosis itself could be considered a predictor of diagnosis in the appropriate clinical context. Mere absence of aneurysms may not exclude PAN. Eleftheriou et al. described a group of 69 children diagnosed with PAN in which nearly 50% were found to have aneurysms on angiography and others had arterial cutoffs and stenoses. None had clinical features of lower limb vascular insufficiency [3].

Also, De Golovine et al. described a patient diagnosed of having PAN with overlapping features of giant cell arteritis who was subsequently found to have peripheral arterial disease on imaging. This patient who is a smoker had presented with features of headache, visual impairment, and abdominal pain without a history of intermittent claudication [12].

Our patient is unique, in the sense that he did not have other risk factors for atherosclerosis and presented with rapidly progressive unilateral intermittent claudication.

## 4. Conclusions

PAN can be a life-threatening vasculitis that may uncommonly present with rapidly progressive intermittent claudication. Early diagnosis with biopsy from a diseased artery and angiography may be useful in diagnosis since a delay of the correct diagnosis could be fatal. The nonaneurysmal segmental stenosis of vessels is an observed variation of vascular involvement in PAN, other than the commonly found aneurysms.
